# Occupational Burnout Prevalence and Its Determinants Among Physical Education Teachers: A Systematic Review and Meta-Analysis

**DOI:** 10.3389/fnhum.2021.553230

**Published:** 2021-12-09

**Authors:** Tariq A. Alsalhe, Nasr Chalghaf, Noomen Guelmami, Fairouz Azaiez, Nicola Luigi Bragazzi

**Affiliations:** ^1^College of Sport Sciences and Physical Activity, King Saud University, Riyadh, Saudi Arabia; ^2^Group for the Study of Development and Social Environment (GEDES), Faculty of Human and Social Science of Sfax, Sfax, Tunisia; ^3^Higher Institute of Sport and Physical Education of Sfax, University of Sfax, Sfax, Tunisia; ^4^Dipartimento di Neuroscienze, Riabilitazione, Oftalmologia, Genetica e Scienze Materno-Infantili (DINOGMI), Genoa University, Genoa, Italy; ^5^Laboratory for Industrial and Applied Mathematics (LIAM), Department of Mathematics, York University, Toronto, ON, Canada

**Keywords:** occupational burnout, physical education teachers, systematic revision and meta-analysis, stress, occupational psychology

## Abstract

Burnout can be defined as an occupational syndrome resulting from poorly managed chronic workplace stress. It is characterized by three dimensions: feelings of energy depletion or exhaustion; increased mental distance from one's job, or feelings of negativism or cynicism related to one's job; and reduced professional efficacy. Teachers are among the human service professionals particularly vulnerable to occupational burnout. Teaching is a highly demanding and challenging task, in that requires constant confrontation with different stakeholders (students and their parents, administrators). Among teachers, physical education teachers have been particularly understudied even though a recently published systematic review has found that they are exposed to high levels of stress. To better explore burnout syndrome among physical education teachers, the present systematic review was undertaken, searching up to six languages. Fifty-six studies were included in the present review. The reported rate of high emotional exhaustion ranged from 11.52 to 60.6%, according to the single study. Pooling together 12 studies and totaling 2,153 physical education teachers, the prevalence rate of high emotional exhaustion was computed to be 28.6 [95% CI 21.9–35.8]. The reported rate of high depersonalization ranged from 3.6 to 45.2%, according to the single study. Pooling together 11 studies and totaling 2,113 physical education teachers, the prevalence rate of high depersonalization was computed to be 14.5% [95% CI 8.0–22.4]. The reported rate of low personal accomplishment ranged from 13.63 to 55.6%, according to the single study. Pooling together 12 studies and totaling 2,153 physical education teachers, the prevalence rate of low personal accomplishment was computed to be 29.5% [95% CI 23.8–35.4]. The reported rate of overall burnout ranged from 10.0 to 51.6%, according to the single study. Pooling together 7 studies and totaling 1,101 physical education teachers, the prevalence rate of overall burnout was computed to be 23.9% [95% CI 13.6–36.0]. No evidence of publication bias could be found, both visually inspecting the funnel plot and conducting the Egger's linear regression test. Burnout imposes a significant burden among physical education teachers. Based on the information contained in the present systematic review and meta-analysis, tailored interventions could be designed to mitigate such a burden. However, due to the limitations of the studies included in the present systematic review and meta-analysis, further research in the field is urgently warranted.

**Systematic Review Registration:** https://osf.io/69ryu/, identifier: 10.17605/OSF.IO/69RYU.

## Introduction

Burnout has been defined in the 11th Revision of the International Classification of Diseases (ICD-11) as an occupational syndrome resulting from poorly managed chronic workplace stress, which is characterized by three dimensions: feelings of energy depletion or exhaustion; increased mental distance from one's job, or feelings of negativism or cynicism related to one's job; and reduced professional efficacy (WHO, 2020).

Teacher burnout has been identified as a worldwide problem (Tsouloupas et al., 2010; Dicke et al., 2015). It has been studied in high or low-income countries of any continents, from North America and Canada to United Kingdom, and from India to Turkey (Ismail et al., 2017). Job satisfaction and teacher burnout have been described in many countries (Batista et al., 2010; Borges et al., 2012; Chennoufi et al., 2012; Converso et al., 2015; Al-Asadi et al., 2018), yet the prevalence of this syndrome in teachers is various (Li et al., 2020).

According to Shirom (1989), the prevalence of burnout in teachers can be situated between 10 and 30%. In Mexican teachers studied by Unda et al. (2008) prevalence of burnout was 17%. Farber found that 5–20% of American teachers are truly burned out (Farber, 1991). Figueiredo-Ferraz et al. (2009) showed a prevalence between 1.9 and 14.20% on the basis of a different profile.

In 35 years of research and practice, burnout has attracted the attention of researchers, practitioners and the general public almost anywhere around the globe, raising many unsolved questions and a significant amount of publications (Schaufeli et al., 2009).

Although there is not a shared definition of Burnout syndrome (BOS), the most widely accepted definition of BOS originates from Maslach and Jackson (1986), who consider this phenomenon as a long-term stress reaction to emotional pressure in human-service professionals, as characterized by a multidimensional model, i.e., emotional exhaustion, depersonalization, and reduce personal accomplishment. These three dimensions of burnout are generally used for any discussion on teacher burnout along with the Maslach Burnout Inventory (MBI)-Educator Surveys, which is a version of the original MBI used for educators, including teachers, and other staff members working in any educational setting (Ghanizadeh and Jahedizadeh, 2015; Mind Garden, 2020).

Emotional exhaustion refers to a depletion of teachers' energetic resources. Hence, in order to cope with emotional exhaustion, teachers develop negative and indifferent attitudes toward their work and, in particular, students (“depersonalization”). Finally, burn-out teachers are likely to perceive themselves as less effective in their job, resulting in feelings of low personal achievement (“reduced personal accomplishment”) (van Horn et al., 1999).

It was found that the three dimensions refer to emotional, cognitive, and behavioral components of burnout (Salmela-Aro and Upadyaya, 2014).

The conceptual framework of the teacher burnout has been explained through the social-exchange theory by Buunk and Schaufeli (1993). According to this theory, the lack of reciprocity in social-exchange relationships at both interpersonal (teacher-student) and organizational (teacher-school) levels is related to higher burnout levels (van Horn et al., 1999).

Therefore, students' misbehavior, as well as poor working condition (e.g., high workload, role conflict and ambiguity, time pressure, inadequate salary and the perceived low status of the profession) can be considered as important stressors in the teaching profession. Moreover, antecedents of burnout have traditionally been divided in 2 categories, i.e., situational and individual antecedents. The first type refers to job and organizational characteristics, the second includes demographic variables (e.g., age, gender, marital status and educational level, work-related attitudes (e.g., high and unrealistic expectations) and personality characteristics (e.g., high level of neuroticism, external locus of control, type A behavior) (Mojsa-Kaja et al., 2015).

According to the Job-Demand Resources Model (Bakker and Demerouti, 2017), work characteristics can be distinguished in job demands and job resources, which can be described at psychological, social, physical and organizational levels. High job demands and low resources can activate the “energetic process” that can lead to BOS. Some work demands such as workload, time pressure, long working hours, and interpersonal conflicts have been found to increase teachers' burnout (Pietarinen et al., 2013).

The Job Demand Resources (JDR) was tested in Finnish teachers, showing that teachers' job demands like pupil misbehavior, workload, and physical work environment can predict BOS, whereas teachers' job resources like job control, supervisory support, information, social climate, and innovativeness, predict positive outcomes such as work engagement and organizational commitment (Hakanen et al., 2006). In another recent research, based on the JDR model, teachers with a higher workload who reported increase in class sizes due to economic circumstances were more likely to belong to engaged-burnout group, whereas teachers who experienced more control over their work and reported higher resilience were more likely to be assigned to engaged group (Salmela-Aro et al., 2019).

Teachers are among the human service professionals particularly vulnerable to occupational burnout. Teaching is a highly demanding and challenging task, in that requires constant confrontation with different stakeholders (students and their parents, administrators).

Among teachers, physical education teachers have been particularly understudied (Brouwers et al., 2011). A recently published systematic review (von Haaren-Mack et al., 2019) has synthesized the prevalence rate of stress, its sources, moderators and consequences among physical education teachers. Authors found that the most relevant sources of stress were the curriculum, the lack of adequate facilities and equipment, the low status of physical education teachers and the students' behaviors and discipline problems. Concerning the consequences and the impact of chronic stress, the majority of physical education teachers indicated overall low-to-moderate levels of burnout, with 20–25% of them reporting high levels of burnout (Sisley et al., 1987; Koustelios and Tsigilis, 2005; Santini and Molina Neto, 2005; De Resende Moreira et al., 2009; Stočkus and Adaškevičiene, 2013; Gama et al., 2014; Sánchez-Oliva et al., 2014; Al Sawy, 2015; Karimi et al., 2015; Shen et al., 2015; Lee et al., 2017; Papasotiriou et al., 2018).

However, the burnout syndrome has been assessed as a consequence of chronic stress and not as a problem *per se*. Indeed, burnout can be considered as an occupational hazard and a specific construct that is different from occupational stress that is framed within the work-related stress hazard. Therefore, burnout can be regarded as both psychosocial hazard and health outcome at the same time (Chirico, 2017; Chirico et al., 2019).

Teachers operate under high levels of stress for significant period of their working time, thus burnout can lead to less sympathy toward students, reduced tolerance and learning by students as well as a lack of commitment and low productivity by teachers (Dorman, 2003).

Therefore, no critical synthesis and appraisal of data concerning burnout among physical education teachers exist. Bearing this in mind, the present study was undertaken in order to fill in this gap of knowledge by providing relevant findings from an extensive investigation in several countries that involves teachers in terms of the prevalence of burnout and its consequences.

## Materials and Methods

### Study Protocol

The study protocol of the present systematic review was devised in accordance to the “Preferred Reporting Items for Systematic Reviews and Meta-Analyses—Protocol” guidelines, and was registered in the “Open Science Framework” (OSF).

### Findings Reporting

Findings of the present systematic review and meta-analysis are reported according to the “Preferred Reporting Items for Systematic Reviews and Meta-Analyses” guidelines.

### Research Questions

The research questions were: (i) which is the prevalence rate of burnout among physical education teachers; (ii) which are the determinants of such as burden.

### Search Strategy

Keywords were searched also in other languages, such as Spanish and Portuguese (“*síndrome da estafa professional*,” “*síndrome de burnout*,” “*desgaste professional*,” “*esgotamento professional*,” or “*exaustão emocional*”), as well as in Farsi/Persian, Turkish, Hebrew and Russian (Table 1).

**Table 1:** Search strategy adopted in the present systematic review and meta-analysis.

**Search strategy item**	**Search strategy details**
Used string of keywords	[(Burnout OR stress OR distress OR exhaustion OR “mental exhaustion” OR “job dissatisfaction” OR “job satisfaction” OR “job motivation” OR “emotional labor” OR “emotional labor” OR emotionality OR “career intention” OR “turnover intention”) AND (”physical education teacher” OR “teacher of physical education" OR “physical education teacher education” OR PETE)]
Bibliographic databases/thesauri	PubMed/MEDLINE, Scopus, SPORTDiscus, PsycNET, ISI/Web of Science, ERIC, and Google Scholar
Inclusion criteria	P: physical education teachers E: exposed to stressors C: age, gender, grade, educational level, ethnicity, etc. O: prevalence rate of burnout and its determinants
Exclusion criteria	P: teachers teaching subjects different from physical education E: not exposed to stressors O: outcomes different from prevalence rate of burnout and its determinants S: reviews, commentaries, editorials
Time filter	None
Language filter	None
Hand-searched target journals	All relevant sports and educational journals

Searched scholarly databases and bibliographic *thesauri* included PubMed/MEDLINE, Scopus, SPORTDiscus, PsycNET, ISI/Web of Science and ERIC. Google Scholar was utilized in order to retrieve the full-text of eligible articles and to screen the gray literature.

Literature screening was done independently by two authors (T.A. and N.C.) and a third author (N.L.B.) acted as final referee in case of disagreements.

Inclusion and exclusion criteria were devised based on the PECOS criteria: P (population), physical education teachers; E (exposure), exposed to stressors and reporting stress/burnout syndrome; C (comparisons/comparators), any kind of comparison (based on age, gender, marital status, educational level, grade, workload, etc.); O (outcomes), the prevalence rate of burnout syndrome and its determinants; and S (study design), any study design (quantitative, qualitative, cross-sectional, longitudinal, etc.).

Similarly, data extraction was carried out by two co-authors. The following data were extracted: surname of the first author, study year, study country, study design, sample size, age and gender distribution, marital status, educational level, instrument used for assessing the burnout, the prevalence rate of the burnout and its determinants (associations/correlations with other constructs). Major findings of each study were also noted.

### Meta-Analysis

Percentage rates were reported using the Clopper-Pearson approach and were synthesized utilizing the Freeman-Tukey method. Heterogeneity among studies was assessed by conducting the *I*^2^ statistics (with amounts of heterogeneity >50% being considered significant). In case of significant heterogeneity, random-effect models were preferred to fixed-effect ones, to better reflect the weight assigned to each included study.

Evidence of publication bias was verified by carrying out the Egger's linear regression test and by visually inspecting the funnel plot. Sub-group analysis and meta-regressions were performed when possible. All meta-analytical computations were performed by means of the commercial software “Comprehensive Meta-Analysis” (CMA for Windows, version 3.0) and the graphs were generated by means of the commercial software MedCalc.

## Results

The initial literature search resulted in a pool of 450 results. After screening titles and/or abstracts and checking for duplicates, 56 studies were included in the present systematic review and literature.

### Instrument Utilized

Various definitions of burnout exist: for instance, Freudenberger (1989) defined burnout as a state characterized by frustration and/or fatigue arising by the lack of the expected reward, whereas, based on the definition of Pines and Aronson (1983), burnout can be conceived as a state of high and intense physical, emotional and mental exhaustion deriving by a long-term engagement in emotionally demanding and challenging situations. According to Cherniss (1986), burnout is a 4-dimension concept, characterized by weariness (that can be internal, exhaustion, or external, aloofness) and discontent (that can be internal, self-dissatisfaction, or external, deprecation). According to Jackson et al. (1986), Maslach and Jackson (1986), Maslach and Schaufeli (1993), and Maslach et al. (2001), burnout is a complex phenomenon, characterized by emotional exhaustion, depersonalization, and perception of low personal accomplishment. These authors have proposed the theoretical framework of burnout most commonly employed in the scholarly literature and have developed the most utilized instrument, the “Maslach Burnout Inventory” (MBI). According to Boyko (2008), emotional burnout can be defined as the consequence of an array of mechanisms and protective strategies, partially filtered by personality, when the individual is exposed to various psycho-traumatic situations. The first phase of emotional burnout is characterized by tensions due to instability of circumstances and psycho-traumatic factors/stressors. The second phase is characterized by resistance, which is followed by moments of jitteriness and, finally, by exhaustion. The theoretical framework utilized for the development of this inventory is a combination of the Selye's theory of general adaption syndrome and the Maslach's theory of burnout syndrome. According to Bakker et al. (2014), burnout refers to a state of exhaustion and cynicism toward work. Even though these conceptualizations may capture different aspects of burnout, all of them share the definition of burnout as a state of elevated fatigue and emotional strain, resulting from disillusionment (Pines, 1993).

Most studies utilized the MBI, with some investigations using the *ad hoc* modified version for educators, teachers, administrators and staff members (MBI-ES). In some cases, the MBI-General Survey (MBI-GS) was employed. MBI consists of 22 items, covering the areas of emotional exhaustion (9 items), depersonalization (5 items) and personal accomplishment (8 items). MBI-GS has a slightly different structure (16 items), with a shorter scale for the measurement of the exhaustion (5 items) and two scales assessing the cynicism (5 items) and the professional efficacy (6 items), which replace the scales of depersonalization and personal accomplishment.

Fejgin et al. (1995) used the Friedman's Burnout Index, which was developed based on the Cherniss' conceptual framework of burnout. Few studies employed the Pines and Aronson's Burnout Index, which, differently from the MBI, enables researchers to quantify the level of burnout by means of a single overall score and is not specific for an occupational field. This burnout measure has been criticized by some researchers in terms of phrasing and dimensionality of the construct.

Finally, Cherepov et al. (2018) employed the Boyko's Emotional Burnout Inventory.

### Study Design

Most studies were cross-sectional, quantitative questionnaire-based investigations, with few studies being qualitative and one study being longitudinal (Table 2).

**Table 2:** Main characteristics of included studies.

**References**	**Study design**	**Country**	**Instrument**	**Sample size**	**Education level**	**Age**	**Gender**	**Teaching experience**	**Marital status**	**Burnout scores**
Both and do Nascimento (2010)	Cross-sectional study	Brazil	MBI	44	NR	NR	NR	NR	NR	•High emotional exhaustion 16.7 % •High depersonalization 45.2% •Low personal accomplishment 26.2 %
Bremm et al. (2017)	Cross-sectional study	Brazil	MBI	9 (55.6% teaching in private schools, 33.3% teaching in public schools, 11.1% teaching in both schools; 11.1% teaching 1 h per week, 22.2% teaching 10–20 h per week, 22.2% teaching 21–30 h per week, 33.3% teaching 31–40 h per week, 11.1% teaching more than 40 h per week; 22.2% teaching at primary schools, 22% teaching at high schools, 55.6% teaching both at primary and high schools)	33.3% with degree, 66.6% with bachelor degree; 88.9% with a post-degree formation, 11.1% without a post-degree formation	20–51 years (44.4% in the 20–40 years age-group, 55.5% in the 41–60 years age-group)	44.4% female, 55.6% male	22.2% with teaching experience of 1–9 years, 33.3% with teaching experience of 10–20 years, 44.4% with teaching experience of more than 20 years	44.4% single, 44.4% married	•Emotional exhaustion 18.00 ± 13.16 •Depersonalization 4.35 ± 2.51 •Personal accomplishment 40.30 ± 7.12 •High emotional exhaustion 33.3% •High depersonalization 22.2% •Low personal accomplishment 55.6% •Overall burnout-rate 22.2%
Brudnik (2010)	Cross-sectional study	Poland	MBI	62	NR	NR	46.8% male, 53.2% female	NR	NR	Overall burnout 51.6%
Cherepov et al., 2018	Cross-sectional study; representative sample	Russia	Boyko's Emotional Burnout Inventory	200	NR	60% aged older than 45 years	90% female, 10% male	7% with experience less than years, 6% with experience <10 years, 24.5% with experience <20 years, 23% with experience <25 years, 39.5% with experience >25 years, 60% with experience of 20 years minimum	NR	•Overall burnout 21% (29% including also borderline cases) •Tension 30% Resistance 34% •Exhaustion 15%
Coterón and Franco (2019)	Cross-sectional study with convenience sample	Spain	MBI	28	NR	39.43 ± 9.08 years (27–59 years)	50% female	NR	NR	•Emotional exhaustion 3.11 ± 1.14 •Depersonalization 2.37 ± 0.92 •Personal achievement 5.88 ± 0.68 •Burnout 33.3%
da Rocha Pimentel et al. (2013)	Cross-sectional study	Brazil	MBI-HSS version	40	NR	42 years	53.8% female	NR	NR	•High emotional exhaustion 50% •Low personal accomplishment 48%
Farsani et al. (2012)	Cross-sectional study	Iran	MBI	250	NR	28–49 years	44% female	NR	NR	•Emotional exhaustion 19.45 ± 10.12 •Depersonalization 4.73 ± 3.34 •Personal accomplishment 32.18 ± 10.34 •High emotional exhaustion 19.45% •High depersonalization 4.73% •Low personal accomplishment 32.18%
Guedes and Gaspar (2016)	Cross-sectional study with *a priori* sample size power analysis (representative sample)	Brazil	MBI	588 (49.7% teaching in primary schools, 49.8% with work hours/workload of 21–40 hours/week, 39.6% working in 2 places, 34.5% with a financial gain of 3–4 minimum wage/month)	33.2% with a degree 52.9% with a specialization	37.7% age ≤30 years	46.4% female	teaching/working experience of 11–20 years	NR	•Emotional exhaustion 31.82 ± 6.87 •Depersonalization 10.57 ± 3.40 •Personal accomplishment 45.48 ± 7.39 •Burnout 10.2% •High emotional exhaustion 27.5% •High depersonalization 27.9% •Low personal achievement 29.9%
Ha et al. (2011)	Cross-sectional study	South Korea	MBI	132 out of 300 teachers of secondary schools (58.3% middle schools, 62.9% public schools; 44.8% with a dual role)	NR	37.94 ± 9.25 years	37.1% female, 62.9% male	11 ± 8.35 teaching experience years	73.9% married, 17.9% single, 5.2% divorced/separated	•Emotional exhaustion 26.40 ± 14.90 •Depersonalization 13.00 ± 8.44 •Personal accomplishment 29.28 ± 11.00 •High emotional exhaustion 26.4% •High depersonalization 13% •Low personal accomplishment 29.28%
Martin et al. (1999a,b)	Cross-sectional study	USA	MBI	294 (60% working as athletic directors, the remaining with further duties and responsibilities)	NR	45.8 ± 8.6 years old (range 22–65 years)	100% male	NR	NR	•Emotional exhaustion 2.6 ± 1.2 •Depersonalization 1.9 ± 1.1 •Personal accomplishment 4.6 ± 0.9 •High emotional exhaustion 36.2% •High depersonalization 17.4% •High personal accomplishment 16.8%
De Resende Moreira et al. (2009)	Cross-sectional study; convenience sampling	Brazil	MBI	149 (out of an initial list of 157 subjects)	Graduated 40.5%, post-graduated 59.5%	Aged 24–34 years 32.7,%, 35–45 34.5%, 46–56 29.3%, >57 years 3.4%	56.4% female	Less than 10 years of teaching experience 67.6%, 10–20 years 24.1%, more than 20 years 8.3%	Married 60.8%, non-married 39.2%	•High emotional exhaustion 36.9% •High depersonalization 16.1% •Low professional accomplishment 16.8%
Ölmez et al. (2018)	Cross-sectional study	Turkey	MBI	76	NR	NR	NR	NR	NR	•High emotional exhaustion 11.52% •High depersonalization 4.13% •Low personal accomplishment 13.63%
Pires et al. (2012)	Cross-sectional study	Brazil	MBI	40	NR	NR	NR	NR	NR	•High emotional exhaustion 17.6% •High depersonalization 3.6% •Low personal accomplishment 36.1%
Sinott et al. (2014)	Cross-sectional study	Brazil	MBI-ES	94	Graduate 52.1%	Less than 40 years 46.8%, more than 40 years 53.2%	63.8% female	NR	Married 54.3%, non-married 45.7%	•High emotional exhaustion 60.6% •High depersonalization 22.3% •Low personal accomplishment 34%
Smith and Leng (2003)	Cross-sectional study; pilot study on 10 subjects	Singapore	Pines and Aronson's Burnout Index	74	NR	6.8% aged 21–24 years, 25.7% aged 25–29 years, 23.0% aged 30–34 years, 20.3% aged 35–39 years, 9.5% aged 40–44 years, 14.9% aged 45 years and older	74.3% male, 25.7% female	58.1% married, 41.9% single	43.2% with < years of experience, 18.9% with 5–9 years of experience, 23.0% with 10–14 years of experience, 14.9% with more than 14 years of experience	•Overall burnout index 3.01 ± 0.98 (range 1.05–5.24) •Burnout 20%
Tsigilis et al. (2011)	Cross-sectional study	Greece	MBI	437 (207 teaching in primary schools, 230 teaching in secondary schools)	NR	40.21 ± 4.19 years teaching in primary school; 42.57 ± 5.89 years teaching in secondary schools	44.4% female in primary schools; 40.9% female in secondary schools	NR	Teaching experience 11.91 ± 4.85 years in primary schools; 15.44 ± 7.81 years in secondary schools	•Emotional exhaustion 15.44 ± 8.67 •Depersonalization 3.60 ± 4.11 •Personal accomplishment 39.34 ± 6.42 •High emotional exhaustion 17.2% •High depersonalization 3.85% •Low personal achievement 38.8%
Viloria et al. (2003)	Cross-sectional study	Venezuela	MBI	140 (teaching in national schools 62.9%, n state schools 27.1%, in private schools 10.0%; teaching at second level (basic) 52.9%, teaching at third level (basic) 34.3, teaching in professional schools 12.8%)	With a bachelor 20.7%, with a degree 74.3%, with specialization 5.0%	Aged <30 years 17.1%, aged 30–40 years 51.4%, aged more than 40 31.0%	49.3% female	Teaching experience years <10 years 53.5%, 10–20 years 27.9%, more than 20 years 18.6%	Married 72.1%, single 18.6%, divorced 4.3%	High burnout level 10%

### Prevalence Rate of Burnout

#### Prevalence Rate of High Emotional Exhaustion

**Figure 1:**
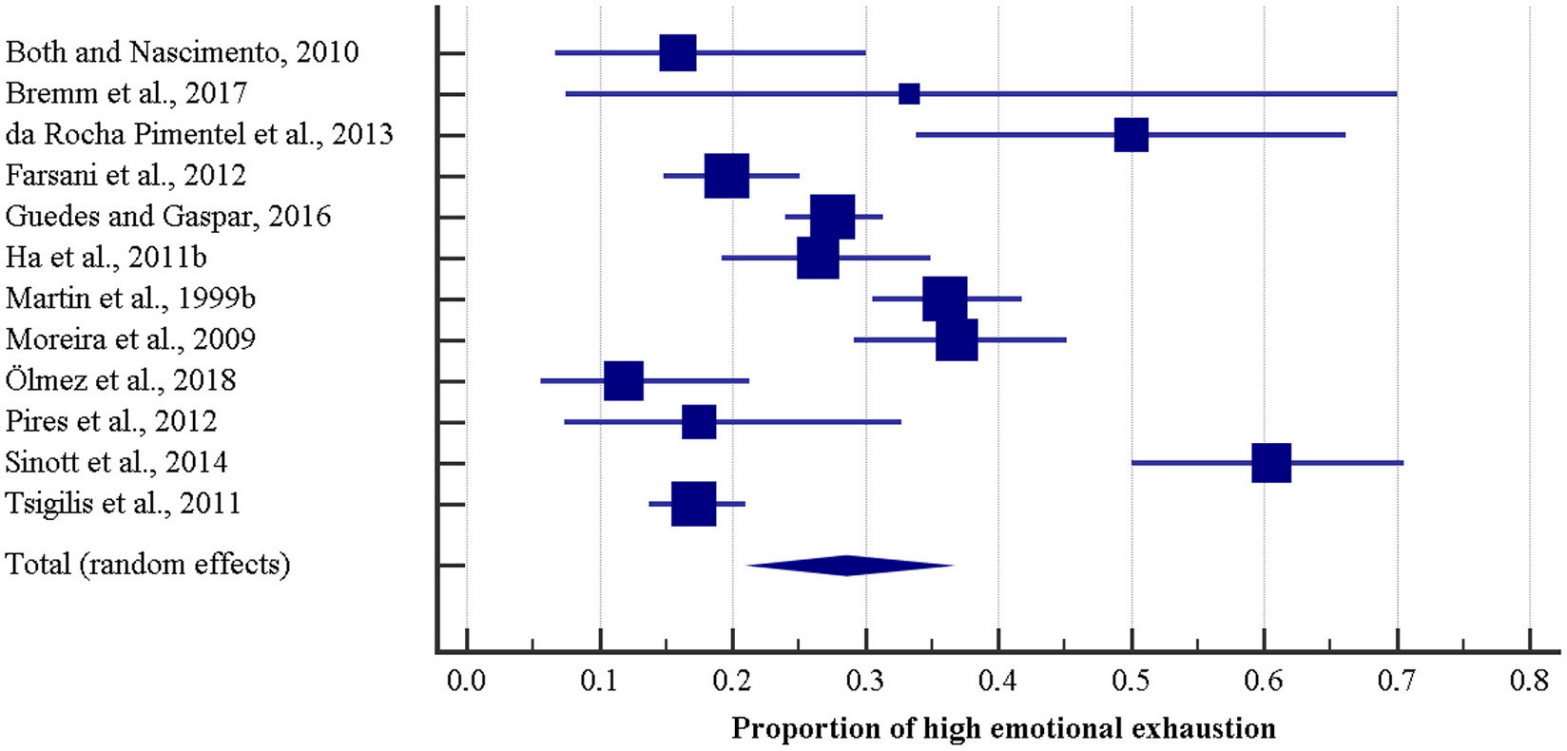
Forest plot of the prevalence rate of emotional exhaustion among physical education teachers.

The reported rate of high emotional exhaustion ranged from 11.52 to 60.6%, according to the single study (Q = 120.72, DF = 11, significance level *p* < 0.0001, *I*^2^ = 90.89% [95% CI 86.01–94.06]). Pooling together 12 studies and totaling 2,153 physical education teachers, the prevalence rate of high emotional exhaustion was computed to be 28.6% [95% CI 21.9–35.8] (Figure 1).

**Figure 2:**
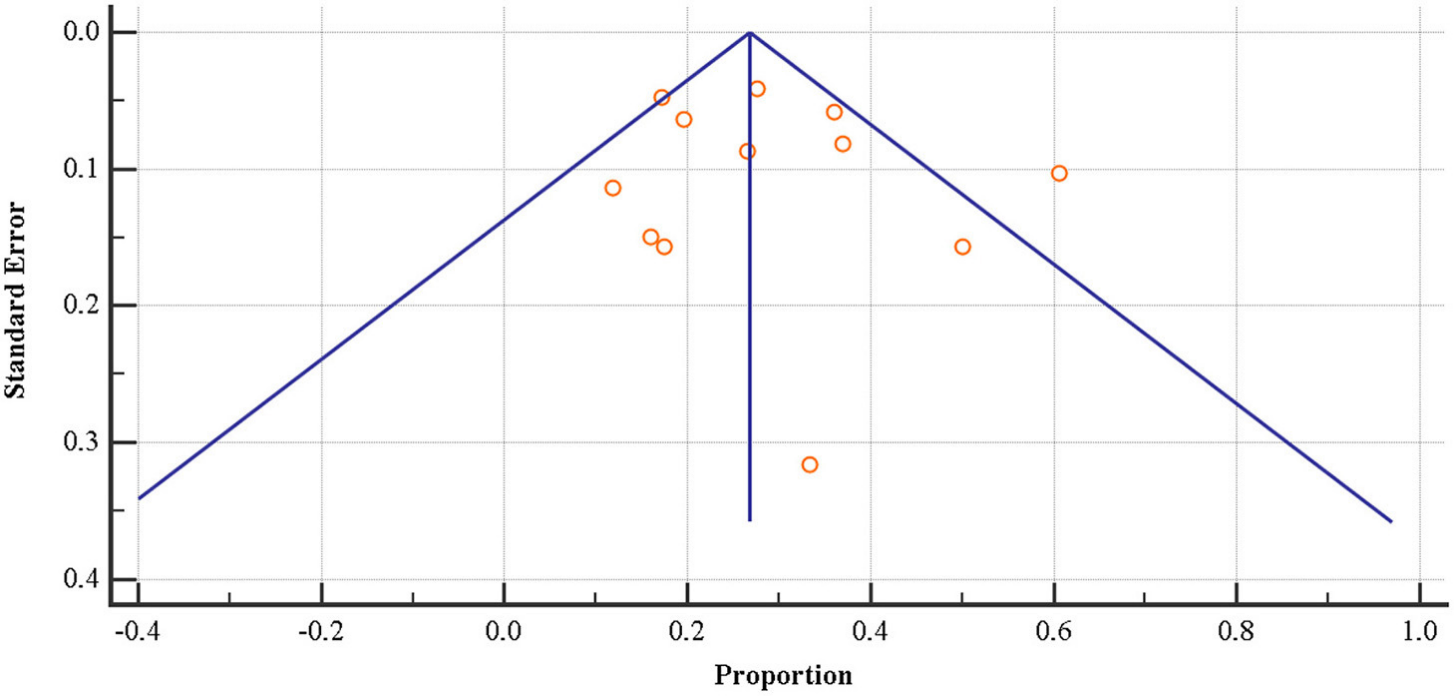
Funnel plot of the prevalence rate of emotional exhaustion among physical education teachers.

No evidence of publication bias could be found, both visually inspecting the funnel plot (Figure 2) and conducting the Egger's linear Regression test.

#### Prevalence Rate of High Depersonalization

The reported rate of high depersonalization ranged from 3.6 to 45.2%, according to the single study. Pooling together 11 studies and totaling 2,113 physical education teachers, the prevalence rate of high depersonalization was computed to be 14.5% [95% CI 8.0–22.4]. No evidence of publication bias could be found, both visually inspecting the funnel plot and conducting the Egger's linear regression test.

#### Prevalence Rate of Low Personal Accomplishment

The reported rate of low personal accomplishment ranged from to, according to the single study. Pooling together 12 studies and totaling 2,153 physical education teachers, the prevalence rate of low personal accomplishment was computed to be 29.5% [95% CI 23.8–35.4]. No evidence of publication bias could be found, both visually inspecting the funnel plot and conducting the Egger's linear regression test.

#### Prevalence Rate of Overall Burnout

The reported rate of overall burnout ranged from 10.0 to 51.6%, according to the single study. Pooling together 7 studies and totaling 1,101 physical education teachers, the prevalence rate of overall burnout was computed to be 23.9% [95% CI 13.6–36.0]. No evidence of publication bias could be found, both visually inspecting the funnel plot and conducting the Egger's linear regression test.

### Determinants of Burnout

Several predictors of burnout have been explored in the existing scholarly literature. The following paragraphs will provide the readers with a comprehensive list of associations found.

#### Age

In the work of Brouwers et al. (2011), age was a predictor of emotional exhaustion and personal accomplishment but not of depersonalization, which resulted, instead, predicted by age in the paper by Carraro et al. (2010). Age was found to be a determinant of personal achievement in the work by Colakoglu and Yilmaz (2014) and Chalghaf et al. (2019). Danylchuk (1993) found that age predicted emotional exhaustion. Esslinger, Guedes and Gaspar, and Viloria reported age to be a predictor of burnout, while Özşaker (2012) identified age-group as a predictor of high desensitization (being higher in the 41–50 years age-group) and of personal achievement. Sinott et al. (2014) found a significant impact of age on personal accomplishment, a finding that was replicated by Spittle et al. (2015). According to Walter et al. (2009), age was a predictor of emotional exhaustion and depersonalization, being higher among younger subjects.

#### Gender

Gender represents an important socio-demographic variable that has been found to inconstantly correlate with burnout. For example, Guedes and Gaspar (2016) reported that being male was associated with experiencing higher levels of burnout. Similarly, in the study by Ahmadian et al. (2015), gender was found to be a predictor of depersonalization, with this sub-scale being higher among males. A similar trend was found by Bremm et al. (2017), whereas in the study by Both and do Nascimento (2010), gender was a predictor of burnout, with females experiencing more burnout than males. The latter finding was replicated by Carraro et al. (2010), who reported that gender was a predictor of emotional exhaustion and personal accomplishment, with higher burnout levels among females. Similarly, Niazi et al. (2014) found that females reported higher scores of emotional exhaustion and depersonalization, and lower scores of reduced personal success with respect to their male counterpart. Interestingly, Brudnik found that gender was a determinant of burnout in a unique way: female teachers tended to react to arrogant behavior, disobedience and disrespect with a negative sense of professional accomplishment, while male teachers reacted with depersonalization. Whereas, Walter et al. (2009) and Panagopoulos et al. (2014) were able to find an association between gender and burnout (emotional exhaustion), in the other studies, on the contrary, gender was not associated with burnout.

#### Marital Status

Danylchuk (1993) found that marital status (in particular, being single) was a predictor of burnout. Yilmaz (2018) was able to replicate such a finding, with marital status (being single) being a predictor of emotional exhaustion, depersonalization and the overall burnout scale score. Finally, according to Panagopoulos et al. (2014), family status was a predictor of personal accomplishment.

#### Professional Qualification/Educational Level

Qualification/educational level was a determinant of predictor in the study by Guedes and Gaspar (2016), where teachers with specialization and graduation experienced higher levels of burnout. Similarly, Karakaya et al. (2015) found that educational background was a determinant of personal failure. The investigations by Vieira et al. and Viloria et al. replicated such findings. Yilmaz (2018) found a correlation between educational level and the total burnout score.

#### Seniority/Years of Teaching Experience

Yilmaz (2018) was able to find a correlation between burnout and years of teaching experience. Olmez et al. found that teaching experience was, in particular, a predictor of emotional exhaustion and personal success.

#### Educational Stage and Type of School

Educational stage is another variable that has been found associated with burnout in a number of studies. For example, Both and do Nascimento reported that teaching in elementary school vs. kindergarten correlated with higher burnout levels. This association could be found for depersonalization, but not for emotional exhaustion or personal accomplishment. Tsigilis et al. (2011) found that educational stage was a predictor of burnout, with lower burnout among educators teaching in secondary schools vs. those teaching in primary schools.

Kroupis et al. (2017) reported that school sector (public vs. private) was a predictor of emotional exhaustion.

#### Type of Teacher

Shirazi et al. (2011) found important differences in terms of official vs. tuitional teachers. More in detail, the frequency and intensity of emotional exhaustion among official teachers was 8.55 and 3.11% more than among tuitional teachers, respectively, whereas the frequency and intensity of reduced personal achievement among official teachers was 13.22 and 19.68% less. The frequency and intensity of depersonalization among official teachers was 22.86 and 11.33% less than tuitional teachers.

#### Working, Organizational, and Environmental Conditions

Working and organizational/environmental conditions represent the variable that has been found to be associated with burnout by the highest number of studies. Yilmaz (2018) found an association between schools' conditions and burnout syndrome. Smith and Leng (2003) reported a correlation between burnout and various work environment dimensions (psychological, *r* = 0.401, social, *r* = 0.400, and bureaucratic, *r* = 0.496). Bai (2014) found that organizational climate of the school was a predictor of burnout (*r* = 0.384). According to Quigley et al. (1989) size of school, amount of administration support for coaching, and the compensation, recognition, and rewards for coaching were correlated with burnout. Bentzen et al. (2014) reported that working/environmental conditions (including heavy workloads, a lack of leader support, and work-related conflicts) were significant determinants of burnout. Similarly, Fejgin et al. (2005) described correlations between burnout and work environment dimensions (psychological, *r* = 0.151, social, *r* = 0.275, and bureaucratic *r* = 0.276). In particular, remuneration, bureaucratic limitations, role limitations were among the factors being most associated with burnout. Moreira et al. reported that working conditions, availability of human resources, social support and inclusion, and organizational aspects correlated with emotional exhaustion. Work growth opportunities and relationship with daily life correlated with emotional exhaustion, depersonalization and professional achievement, whereas work-related health quality of life correlated with emotional exhaustion and professional Achievement. Cieśliński and Szum (2014) found that bad working conditions, subjectively perceived low salary, and mismanagement of education as well as the necessity to work with big groups of students (socially maladjusted) and didactic and/or educational challenges were determinants of burn-out syndrome. Kroupis et al. (2019) found that the level and conditions of school sports facilities were a predictor of emotional exhaustion. Finally, Guedes and Gaspar (2016) found that workplace related aspects (working in ≥3 places), line of work (physical conditioning and primary school), financial gain (≤2 minimum wage) and work hours/workload (≥41 h/week) were predictors of burnout.

#### Dual Role Conflict

Few investigations have focused on the stress perceived by physical education teachers who are also coaches and, as such, can experience the role stress and strain due to the challenging and often conflicting demands deriving from their peculiar condition. For instance, Iannucci and MacPhail (2018) have found a significant impact of perceived role conflict on burnout syndrome. Kelley and Gill (1993) have reported that the teacher-coach role conflict is a determinant of burnout.

#### Students' Behavior

Coterón and Franco (2019) found a mutual relationship between burnout syndrome and student's behavior. This was confirmed by the study by Karakaya et al. (2015), which found that student athletes' behavior (indiscipline, such as speaking without permission during the course/lesson, not having the necessary equipment for the course/lesson, not performing a given task and not listening to the teacher) was a predictor of burnout. Also in the article of Sawy, Students' indiscipline and behavioral issues were predictor of burnout. Finally, Yilmaz (2018) reported an association between the lack of interest of students, not giving adequate importance to lessons, and teachers' burnout syndrome.

#### Parental Conflicts

Only one study (Esslinger) analyzed the potential impact of parental conflicts on teachers' burnout, finding that, despite the expectations and despite the increase of such conflicts throughout the time of the study, they were not associated with burnout.

#### Correlation Between Burnout and Job Satisfaction

In the study by Ahmadian et al. (2015), burnout levels correlated with job satisfaction (*r* = −0.331 for emotional exhaustion, *r* = −0.353 for depersonalization, and *r* = 0.618 for personal success). In the work by Nascimento, burnout impacted on quality of life at the workplace and job satisfaction (*r* = 0.35). Similar, Ciris et al. found a significant negative correlation (*r* = −0.531) between job satisfaction and burnout. Job satisfaction was inversely related to burnout in the investigation by Panagopoulos et al. (2014), whereas Ölmez et al. (2018) reported that burnout dimensions and job satisfaction correlated. Finally, Vousiopoulos et al. (2019) found a negative association between burnout and job satisfaction.

#### Correlation Between Burnout and Commitment

Shirazi et al. (2011) found that there was a relationship between normative commitment and depersonalization among official teachers, while there was an association between normative, affective commitment and depersonalization among tuitional teachers.

#### Correlation Between Burnout and Other Constructs

Niazi et al. (2014) reported that burnout correlated with mental health, whereas, according to Carraro et al. (2010), personal accomplishment correlated with the self-perception of physical fitness, and the perception of the influence of personal fitness on teaching. Interestingly, Farsani reported correlations between burnout and teachers' personality: extraversion negatively correlated with emotional exhaustion, openness to experience negatively correlated with emotional exhaustion, depersonalization and positively correlated with personal accomplishment, agreeableness negatively correlated with emotional exhaustion, depersonalization and personal accomplishment, neuroticism positively correlated with emotional exhaustion, depersonalization and personal accomplishment, while general health negatively correlated with emotional exhaustion, depersonalization and personal accomplishment.

#### Teaching Adapted Physical Activity

Very few studies have assessed burnout among physical educators dealing with adapted physical activity.

## Discussion

To the best of our knowledge, the present systematic review and meta-analysis represents the first and most comprehensive assessment of burnout syndrome and its determinants among physical education teachers.

Our study shows that the burden is relevant, even though highly variable among the studies. This high variability and heterogeneity could be explained taking into account country-specific features, depending on the importance assigned to physical education in a given setting and the rewards assigned to physical educators. Differences in samples recruited in terms of gender and age distribution, marital status, ethnicity, highest educational level attained, can also contribute to explain, at least partially the amount of heterogeneity.

A variability was also noted in terms of sources of stressors to which physical education teachers are exposed and the determinants of burnout syndrome, with most findings being inconsistent (in terms of values and direction of the impact on stress and burnout) and difficult to be replicated. Working and environmental factors seem to be those which have been replicated by most studies, even though with different protocols and different theoretical frameworks/methodologies.

Despite such heterogeneity, burnout among physical education teachers represents a global public health concern and health decision- and policy-makers should devise and implement *ad hoc* interventions and measures, based also on the findings of studies reported at country level.

Burnout significantly impacts on perceived quality of life of teachers and may contribute to their choice of leaving the job. Physical activity is a major non-pharmacological tool for keeping the physical and mental fitness and counteracting several chronic-degenerative disorders and physical education is fundamental for properly educating children and adolescents and avoiding the insurgence of various diseases.

Teacher burnout can affect teaching goals and educational environment, which may contribute to severe problems not only at individual level, but also at organizational context. Indeed, poor job performance, health issues, and adverse student outcomes and other organizational outcomes such as job withdrawal-absenteeism, turnover intention, and actual attrition have been described, thus contributing to lower effectiveness and productivity at work (Maslach et al., 2001; Li et al., 2020).

Despite many strengths (including its methodological rigor, transparency and reproducibility), our systematic review and meta-analysis is not without limitations. The major shortcoming is given by the highly heterogeneous and sometimes insufficient data available, which hindered the possibility of conducting a full, thorough meta-regression analysis, that if performed, could have provided significant insights. Furthermore, some studies included in the present review are characterized by high drop-out rates, which call up for caution when generalizing the findings. Further high quality studies are needed.

## Conclusions

Burnout imposes a significant burden among physical education teachers. Based on the information contained in the present systematic review and meta-analysis, tailored interventions could be designed to mitigate such a burden. However, due to the above-mentioned limitations, further research in the field is urgently warranted.

## Data Availability Statement

The original contributions presented in the study are included in the article/supplementary material, further inquiries can be directed to the corresponding author/s.

## Author Contributions

TA and NB conceived the review. All authors critically contributed to the review.

## Funding

This research was funded by the King Saud University.

## Conflict of Interest

The authors declare that the research was conducted in the absence of any commercial or financial relationships that could be construed as a potential conflict of interest. The reviewer OG declared a shared affiliation, with no collaboration, with one of the authors, NB, to the handling editor at the time of review.

## Publisher's Note

All claims expressed in this article are solely those of the authors and do not necessarily represent those of their affiliated organizations, or those of the publisher, the editors and the reviewers. Any product that may be evaluated in this article, or claim that may be made by its manufacturer, is not guaranteed or endorsed by the publisher.
